# Predictive Value of Microfilariae-Based Stop-MDA Thresholds After Triple Drug Therapy With IDA Against Lymphatic Filariasis in Treatment-Naive Indian Settings

**DOI:** 10.1093/cid/ciae019

**Published:** 2024-04-25

**Authors:** Ananthu James, Luc E Coffeng, David J Blok, Jonathan D King, Sake J de Vlas, Wilma A Stolk

**Affiliations:** Department of Public Health, Erasmus MC, University Medical Center Rotterdam, Rotterdam, The Netherlands; Department of Public Health, Erasmus MC, University Medical Center Rotterdam, Rotterdam, The Netherlands; Department of Public Health, Erasmus MC, University Medical Center Rotterdam, Rotterdam, The Netherlands; Department of Control of Neglected Tropical Diseases, World Health Organization, Geneva, Switzerland; Department of Public Health, Erasmus MC, University Medical Center Rotterdam, Rotterdam, The Netherlands; Department of Public Health, Erasmus MC, University Medical Center Rotterdam, Rotterdam, The Netherlands

## Abstract

Mass drug administration (MDA) of antifilarial drugs is the main strategy for the elimination of lymphatic filariasis (LF). Recent clinical trials indicated that the triple-drug therapy with ivermectin, diethylcarbamazine, and albendazole (IDA) is much more effective against LF than the widely used two-drug combinations (albendazole plus either ivermectin or diethylcarbamazine). For IDA-based MDA, the stop-MDA decision is made based on microfilariae (mf) prevalence in adults. In this study, we assess how the probability of eventually reaching elimination of transmission depends on the critical threshold used in transmission assessment surveys (TAS-es) to define whether transmission was successfully suppressed and triple-drug MDA can be stopped. This analysis focuses on treatment-naive Indian settings. We do this for a range of epidemiological and programmatic contexts, using the established LYMFASIM model for transmission and control of LF. Based on our simulations, a single TAS, one year after the last MDA round, provides limited predictive value of having achieved suppressed transmission, while a higher MDA coverage increases elimination probability, thus leading to a higher predictive value. Every additional TAS, conditional on previous TAS-es being passed with the same threshold, further improves the predictive value for low values of stop-MDA thresholds. An mf prevalence threshold of 0.5% corresponding to TAS-3 results in ≥95% predictive value even when the MDA coverage is relatively low.

Lymphatic filariasis (LF), a neglected tropical disease (NTD), is a leading cause of preventable morbidity and disability due to lymphedema, hydrocele, and acute inflammatory episodes with resultant fevers (acute dermatolymphangioadenitis) and still affects more than 50 million people worldwide [1]. The most common causative agent is the parasitic filarial nematode worm *Wuchereria bancrofti*. Adult worms are found in lymph vessels, whereas the worm’s offspring microfilariae (mf), which are released by fertilized female worms, are picked up from blood and transmitted to humans by mosquitoes. The Global Programme to Eliminate Lymphatic Filariasis was initiated in 2000 with the aim of interrupting the transmission of LF by implementing mass drug administration (MDA) with the 2-drug combinations of diethylcarbamazine and albendazole (DA) in onchocerciasis-free areas or ivermectin and albendazole (IA) in areas where onchocerciasis prevails [2, 3]; where loiasis is present, twice yearly albendazole is the recommended treatment regimen [3]. This is to be combined with improved morbidity management to alleviate the suffering of people with clinical manifestations.

Recent clinical evidence indicated that MDA with a triple-drug regimen of ivermectin, diethylcarbamazine, and albendazole (IDA) is even more effective than the 2-drug regimens [4–7], leading to the World Health Organization (WHO) recommendation in 2017 of using IDA for LF control in LF-endemic areas not co-endemic with either onchocerciasis or loiasis [8]. This includes India, where approximately 55% of the current global burden of LF is located, with approximately 487 million people at risk of infection [3]. MDA was initiated in 2004 in 202 Indian districts with diethylcarbamazine and in 2007 in all 256 endemic districts at that time with DA [9]. In 2018, IDA was introduced in 5 districts that either failed a transmission assessment survey (TAS) or never undertook MDA despite the identification of LF transmission. This was followed by the selection of 21 additional districts for IDA implementation in the next year. In 2020, 16 districts were newly classified as endemic for LF, increasing the total number to 272.

MDA programs that use the various drug regimens have successfully reduced LF prevalence in many affected areas so that the infection is either eliminated as a public health problem (mf prevalence <1% or antigen prevalence <2%) or elimination is close to being achieved. To save resources and time, it is important to establish threshold prevalences below which MDA can be stopped as soon as possible but with minimal risk of disease resurgence. For areas treated with a 2-drug regimen, the stop-MDA decision is made based on TAS-1 that assesses the prevalence of circulating filarial antigenemia (CFA) in children aged 6–7 years [10]. Elimination is considered validated if antigenemia prevalence in this age group is sustained below the predefined threshold in two subsequent TAS-es (TAS-2 and TAS-3), with intervals of two years minimum between surveys. However, compared with 2-drug regimens, IDA leads to a faster mf clearance and potential sterilization of adult worms, resulting in fewer required treatment rounds. This means that after the last MDA round, antigens are likely to persist [11]. WHO recognizes that new diagnostics are needed, and target product profiles have been developed [12, 13]. Until such new tests are available, testing for mf is the best way to identify persons with reproducing adult worms. Because adults, in contrast to children, are the most likely to be mf-positive and have the lowest MDA coverage, it might be useful to target TAS in IDA-treated areas at adults [14].

Mathematical models have been helpful in assessing the validity of stop-MDA thresholds [15, 16]. Here, we use the already established LYMFASIM model [16] to simulate IDA-MDA in treatment-naive Indian settings and to assess the probability of achieving elimination of LF transmission in relation to the chosen mf-based stop-MDA threshold used in TAS-1, TAS-2, and TAS-3. To understand the impact of the treatment regimens used, including the uncertainty regarding the effect of IDA on adult worms [17], we compare the results for MDA with IDA and DA. We identify situations for which predictive values as high as 95% are possible.

## METHODS

We adopted the same methods that were used in a previous publication on assessing stop-MDA thresholds for the African context [16]. To simulate population-level LF transmission dynamics, we used the individual-based LYMFASIM model, which accounts for interhost variation in exposure to transmission and uptake of MDA. The model was quantified for Indian settings using data from approximately 25 000 individuals from Pondicherry in 1981 and longitudinal measurements of human infection status in 1981, 1986, 1989, and 1991 [18]. This Indian model variant includes a host immunity mechanism that regulates parasite establishment [18, 19], which is absent for the African version of LYMFASIM [16]. As a result, transmission can better maintain itself at low levels without incoming infections from neighboring areas. For the analysis presented here, we simulated a single community with a population of approximately 1000 people.

We performed simulations for a range of baseline mf prevalences between 5% and 20%, assuming that simulated communities underwent no previous treatment. Baseline mf prevalences were determined using two transmission parameters: the monthly biting rate and the shape of the gamma distribution that describes exposure variation between individuals. We repeatedly sampled values for these two parameters from a predefined parameter space (Supplementary Figure 1); individual sets of parameter values were adopted until we had 250 parameter combinations for each 1%-wide bin between 5% and 20% (ie, 15 bins). Next, we simulated the impact of 3–6 rounds of annual MDA using either IDA or DA, implemented at either 65% or 80% of the total population and comprising all age groups (ie, 16 MDA scenarios). We assumed the macrofilaricidal effect of IDA to be the same as that of DA (55%) but with 100% mf killing and permanent sterilization of female adult worms (denoted as “IDA1” by Irvine et al. [17]). As such, we ran 3750 simulations per MDA scenario (15 × 250) and 60 000 simulations in total (16 × 3750). Each simulation was marked as having achieved elimination (or not) if the mf prevalence in the entire population was zero (above zero) 20 years after the last MDA round. To evaluate the predictive value of TAS-es for achievement of elimination, we saved the annual post-MDA mf prevalence, starting one year after the last MDA round, for each simulation. We also repeated TAS-1 defining elimination within a 50-year period post-MDA instead of 20.

We evaluated the predictive value of a series of three TAS-es that take place one, three, and five years after the last MDA round, which was first quantified in terms of receiver operating characteristic (ROC) curves. For each LYMFASIM simulation (N = 60 000), we simulated 100 repeated series of TAS-es, assuming a binomial sample of 200 or 400 individuals of ages ≥5 or ≥15 years (ie, four TAS scenarios). For each simulated TAS scenario, varying the stop-MDA threshold, we evaluated the positive predictive values (PPVs; ie, the probability of achieving elimination if the measured prevalence is below the threshold), where a particular threshold value was always used for all TAS-es in the same series. (We did not evaluate negative predictive values since overtreatment was outside the scope of this study.) For TAS-2 and TAS-3, we evaluated the PPV on the condition that previous TAS-es in the same series had been passed. We quantified the impact of TAS(-es) in terms of the maximum increment in PPV as the decision threshold is lowered. Finally, we determined the maximum stop-MDA threshold that achieves a PPV of ≥95%. We repeated this analysis with one of the TAS-es being delayed by two years.

In Supplementary Table 1, we describe our adherence to the five principles of the Neglected Tropical Diseases Modelling Consortium on good practice for policy-relevant modeling [20].

## RESULTS

**Figure 1.**
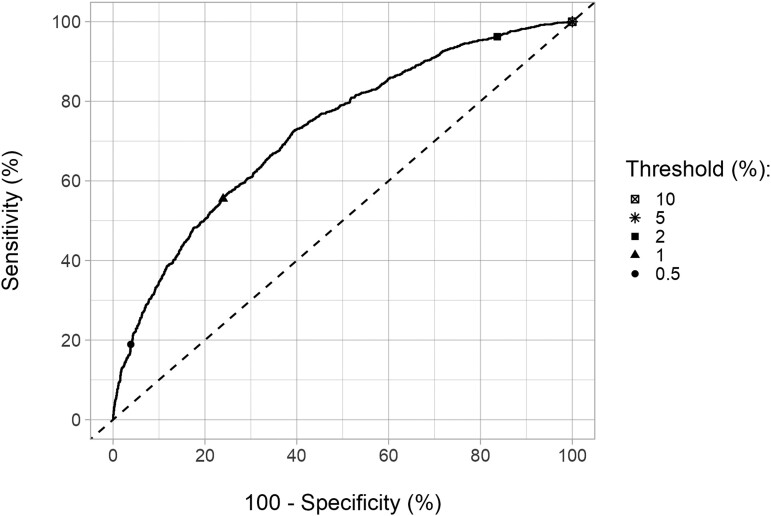
Receiver operating characteristic (ROC) curve at 1-year post–MDA (TAS-1) for age group 5+ under MDA with ivermectin, diethylcarbamazine, and albendazole (IDA). The MDA duration was three years, and coverage was 65%. The ROC curves for a wide range of parameters can be found in Supplementary Figure 2. Note that sampling is not included for the ROC curve(s). Sensitivity (*y*-axis) is the percentage of runs ending in elimination within 20 years post-MDA that are correctly identified based on microfilariae prevalence below a range of thresholds. The *x*-axis, 100%-specificity, represents the percentage of runs falsely classified as having elimination among all the runs that did not result in elimination in the same period.

First, we evaluated the amount of information available from TAS-1 targeting age group 5+ with IDA using the ROC curve (Figure 1). The curve was fairly close to the diagonal, suggesting TAS-1 provides only limited information relative to the *a priori* probability of achieving elimination. Even for a wide range of MDA scenarios, the ROC curves showed similar trends (Supplementary Figure 2). However, the ROC curve for age group 5+ was always further away from the diagonal than age group 15+, indicating that the former is a better option for measuring mf prevalence.

**Figure 2.**
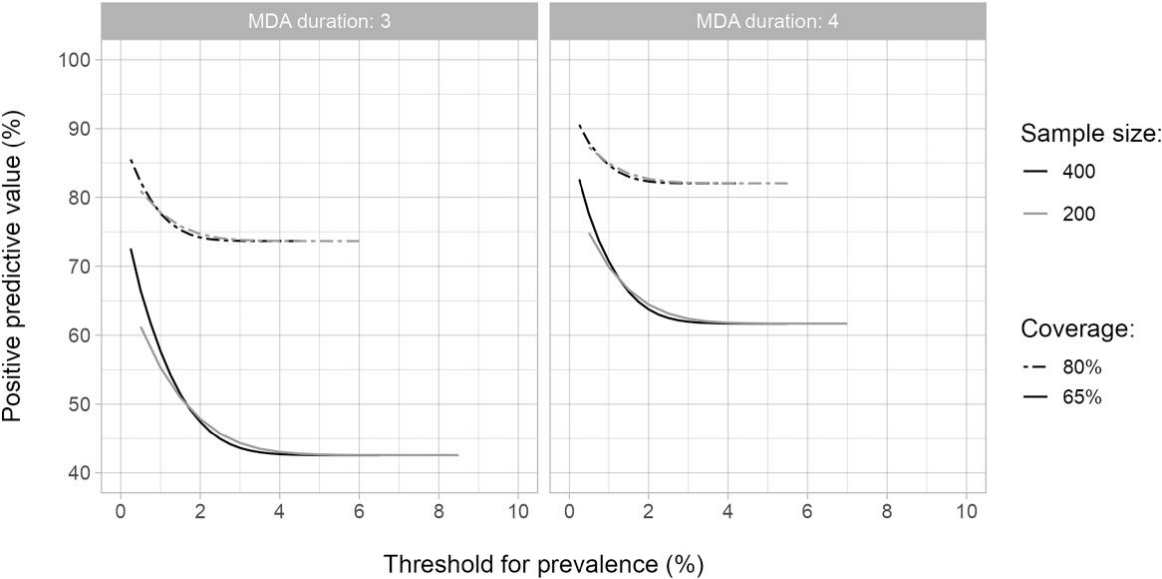
Positive predictive values (PPVs) as a function of stop-MDA threshold for MDA durations three (left panel) and four (right panel) years. PPV was defined as the probability of achieving elimination within 20 years after the last round of IDA-MDA if the microfilariae prevalence 1-year post-MDA (transmission assessment survey-1) was below a given threshold. The results shown are for those aged 5+ years and for two different values of sample sizes (400 and 200) and MDA coverages (65% and 80%). The PPV curves with sample size being 400 for a wide range of other parameters can be found in Supplementary Figure 3.

Next, we evaluated how the PPV of TAS-1 changes as a function of the stop-MDA threshold for mf prevalence in age group 5+ for IDA-treated areas (Figure 2). One common feature of the PPV curves was that as the stop-MDA threshold increased, the PPV decreased to a plateau, equaling the *a priori* probability of achieving elimination across all simulation runs. For IDA-based MDA scenarios, adopting a lower stop-MDA threshold increased the PPV (relative to the plateau) up to approximately 30 percentage-points (Supplementary Figure 3). The corresponding increment associated with DA was approximately 48 percentage-points. Also, sampling 400 instead of 200 individuals increased the maximum PPV by, at most, approximately 10 percentage-points for IDA (Figure 2). As the probability of elimination increased with MDA coverage and duration, so did the PPV; to a degree, this was comparable to the magnitude of increase in PPV when lower stop-MDA thresholds were adopted. Those aged ≥5 years had a higher PPV by, at most, 5 percentage-points compared with those aged ≥15 years for IDA (Supplementary Figure 3). With 80% coverage, PPVs of 85%–95% were possible when the stop-MDA threshold was ≤0.5% mf prevalence, although surveys to assess mf prevalence led to only a small increment in PPV compared with the *a priori* probability of elimination.

For TAS-1, we considered some alternate situations as well. First, given that the exact values of coverage are often unknown in actual settings, we repeated the above analysis by lumping together simulations with 65% and 80% coverage levels (Supplementary Figure 4). The resulting PPV increments were intermediate (with the maximum increments being approximately 25 percentage-points for IDA and approximately 42 percentage-points for DA) between those when the two coverage levels were considered separately. Next, to determine if the predictive power of TAS-1 would improve, we considered elimination within 50 years instead of 20 years (Supplementary Figure 5). The *a priori* probability of elimination was higher if measured 50 years after stopping than if measured 20 years after stopping, resulting in higher absolute PPVs. However, the maximum PPV increments were lower for the former for both drug regimens, indicating its weaker predictive power. The maximum PPV increments were approximately 20 percentage-points and approximately 30 percentage-points for the two periods for IDA-based MDA, whereas they were approximately 35 percentage-points and approximately 48 percentage-points for the DA-based one.

**Figure 3.**
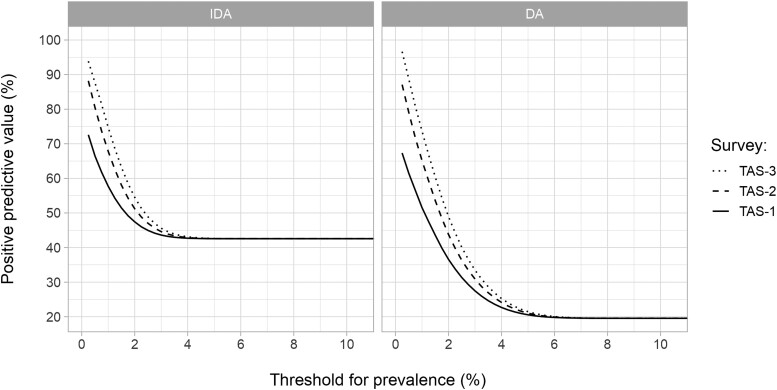
Positive predictive value (PPV) of TAS-1, TAS-2, and TAS-3 for elimination of lymphatic filariasis after three years of mass drug administration (MDA) at 65% coverage using IDA (left panel) or DA (right panel) treatment. Elimination was defined as zero microfilariae prevalence 20 years after the last MDA round. PPVs were calculated as a function of the stop-MDA threshold for prevalence of infection in the age group 5+ (horizontal axis). For TAS-2 and TAS-3, PPVs are conditional on all previous TAS-es being passed with the same prevalence threshold. TAS-1, TAS-2, and TAS-3 were scheduled one, three, and five years post-MDA, so that the gap between consecutive TAS-es was two years. For each TAS, we assumed a sample size of 400.

Finally, keeping the time to achieve elimination as 20 years, we explored the predictive value of TAS-2 and TAS-3, conditional on previous TAS-es being passed (with the same stop-MDA threshold as used in later TAS-es), when based on a sample of ages 5+ (Figure 3). PPV increased with each additional TAS for lower thresholds. This trend remained consistent across all the MDA scenarios that we considered (Supplementary Figure 6). Following TAS-2 and TAS-3, the maximum PPV increments associated with IDA became approximately 45 percentage-points and approximately 52 percentage-points, respectively, compared with the maximum increase of approximately 30 percentage-points for TAS-1 only. The corresponding values for DA were approximately 68 percentage-points and approximately 76 percentage-points, respectively, compared with the approximately 48 percentage-points for TAS-1 only. Thus, PPVs following TAS-3 can be as high as approximately 95 percentage-points for decision thresholds ≤0.5% mf prevalence, even for low values of MDA coverage (65%) and duration (three years).

We also found that delaying TAS-1 by two years so that TAS-es occurred at three, five, and seven years post-MDA had only a limited impact on PPVs (Supplementary Figure 7). The PPV increment was the highest for TAS-1 by approximately 5 percentage-points for both drug regimens. The PPV increments associated with increasing the gap between one of the TAS-es, keeping TAS-1 at one year post-MDA, were even more negligible (not shown).

## DISCUSSION

Our results show that, by itself, TAS-1 conducted to measure mf prevalence one year after the last MDA round with IDA provides relatively little information on the prospect of elimination, which is dictated largely by MDA duration and coverage. However, TAS-2 and TAS-3, conducted three and five years post-MDA (but only if TAS-1 is passed), are more informative in this regard. For TAS-3, PPVs ≥95% are possible with a stop-MDA threshold of ≤0.5% mf prevalence for ages 5 years and above. Further, using a larger sample size for TAS (ie, greater number of individuals) as well as testing the age group of 5+ instead of 15+ increase the PPV of mf surveys. The latter is due to the fact that if MDA successfully suppresses transmission, young individuals are less likely to contract their first worm infection; therefore, in age groups for which prevalence would normally strongly increase with age pre-MDA (ages 5–15 years), a low infection prevalence is indicative of a significant impact on transmission. Including this age group in surveys therefore adds useful information for decision-making [15].

The surveys provide more information in settings with DA than in settings with IDA, which is due to IDA being more effective than DA in clearing LF infection. Results pertaining to DA are helpful in hypothesizing the consequence of an alternate assumption of IDA having no sterilizing effect (denoted as “IDA2” by Irvine et al. [17]), which is contrary to the 100% (permanent) sterilization of female adult worms we considered here. In case of no sterilizing effect, the only difference between IDA2 and DA is IDA2’s ability to kill 100% mf instead of the widely accepted value of 95% mf killing of DA [21]. Therefore, we expect that the results for an analysis of IDA2 would be similar to what we present here for DA.

Previous modeling studies on elimination or stop-MDA thresholds for LF considered 40–50 years after the last MDA round as the time horizon to define elimination (0% mf prevalence) [16, 19, 22], whereas we adopted a time horizon of 20 years. Following the assumption that the parasite numbers are so low by the time MDA is stopped, as per the breakpoint theory [23], the number of new infections post-MDA is not enough to sustain transmission. Hence, when past this breakpoint, a 50-year period would allow for the complete natural attrition of the remaining parasite population in our simulations, resulting in a higher (*a priori*) elimination probability than for a 20-year period. This is why TAS-es added less information in the former case than the latter. More importantly, a 50-year time horizon is far beyond the political scope of most governments and probably not realistic since a lot can happen in 50 years (eg, secular developments or disasters). Although still long, a 20-year time horizon is much closer to the reality in which we develop and use NTD control strategies. For this time horizon, it is more feasible to also compare our model predictions with results from actual settings than from the 50-year period. In real-life settings, before TAS-1, a pre-TAS is often conducted in up to eight communities to determine whether mf prevalence is <1% (CFA prevalence <2%). The purpose of the pre-TAS is to decide whether TAS-1 should be conducted across the evaluation unit (typically a district), which would encompass a large number of communities. As our simulations focus on single communities and not larger areas that consist of multiple communities, we did not account for pre-TAS.

Although testing for mf may be the best way to identify persons with reproducing adult worms, implementing this strategy at scale can be challenging due to the nocturnal periodicity of mf in India and most other countries, with serious cost and resource implications for all programs. Therefore, mf tests will likely not be performed population-wide but will only be done in people who tested antigen-positive in an antigen survey carried out during the day, as in recent studies such as the one by Eneanya et al. [24]. In our study, we did not explicitly consider a pre-screening for CFA and assumed that the probability of an mf-positive individual testing negative on CFA is negligible. Taken together, our results may represent a strategy with decision-making based solely on the estimated mf prevalence. Another concern relates to the limited sensitivity/reliability of mf detection using finger-prick blood in post-treatment settings when parasitemia levels are expected to be low. Our model accounts for this by simulating variation in mf counts, assuming that mf counts follow a negative binomial distribution with aggregation parameter *k* = 0.35. Better sensitivity can be achieved by filtration of larger blood samples, but this is not feasible at a large scale. If the CFA prevalence itself would also be considered in the decision-making (eg, via a second threshold for CFA prevalence), the predictive value of TAS-es might be somewhat higher than we predict here. Further, the timing of TAS-es may not be as we assumed here (every two years, starting one year after the last MDA round). For instance, it is also possible that TAS-es could be delayed in some settings due to external reasons such as coronavirus disease 2019. However, we found such delays to only slightly increase the predictive value of TAS-es, which is reflective of the slow dynamics of LF recrudescence [25].

To assess the status of LF elimination, only mf and CFA prevalences are used as indicators, with the latter not being recommended for IDA-based MDA. For other NTDs, the vector infectivity rate is also often used as an indicator. For example, stop-MDA decisions for onchocerciasis are based on black fly infectivity in addition to antibody seroprevalence [26]. However, in the case of LF, there is no established practice for collecting mosquito infectivity data. This is also because the mosquitoes would need to be captured in many different locations (eg, households) to obtain a representative picture of a community [27], unlike the onchocerciasis-transmitting black flies that circulate throughout a village, making it easy for data to be collected.

In this work, we only considered regions in India that were previously untreated and ignored the impact of other interventions potentially affecting LF infection and transmission, such as the National Deworming Day (NDD) program, initiated in 2015, in which albendazole is administered biannually in those aged up to 19 years [28]. Based on evidence from the Republic of Congo, twice yearly albendazole can strongly reduce antigenemia rates [29, 30], but no information is available yet on the effectiveness of the strategy in the Indian context. Given that a big fraction of the population remained untreated, NDD alone is unlikely to lead to elimination. Still, accounting for this effect could lead to more accurate predictions for areas that are treatment-naive for LF where the NDD strategy is applied. There might also be settings that failed TAS even after multiple rounds of DA and where IDA could be administered for 1–3 rounds, for which a similar analysis would be interesting. IDA can also be administered in LF-endemic African regions that are not endemic to both onchocerciasis and loiasis, such as Madagascar. Our analysis could be extended to such settings as well.

We conclude that when only TAS-1 is included, PPVs are always <95% for threshold values ≥0.5% mf prevalence. However, with two additional TAS-es, spaced two years apart and conditional on all three TAS-es being passed with the same threshold, PPVs of ≥95% are possible for a stop-MDA threshold of 0.5%, even when the coverage is as low as 65%. This study supports the WHO strategy of repeating the TAS twice during post-MDA surveillance, although the PPV could be improved by lowering the decision threshold from approximately 4% to 0.5%.

## Supplementary Data

Supplementary materials are available at *Clinical Infectious Diseases* online. Consisting of data provided by the authors to benefit the reader, the posted materials are not copyedited and are the sole responsibility of the authors, so questions or comments should be addressed to the corresponding author.

## Supplementary Material

ciae019_Supplementary_Data

## Notes

***Supplement sponsorship.*** This article appears as part of the supplement “New Tools and Nuanced Interventions to Accelerate Achievement of 2030 Roadmap for Neglected Tropical Diseases,” sponsored by funding of Professor T. Déirdre Hollingsworth's research by the Li Ka Shing Foundation at the Big Data Institute, Li Ka Shing Centre for Health Information and Discovery, University of Oxford; and funding of the NTD Modelling Consortium by the Bill & Melinda Gates Foundation (INV-030046).

## References

[1] Local Burden of Disease 2019 Neglected Tropical Diseases Collaborators . The global distribution of lymphatic filariasis, 2000–18: a geospatial analysis. Lancet Glob Health 2020; 8:e1186–94. 32827480 10.1016/S2214-109X(20)30286-2 PMC7443698

[2] Ottesen EA, Hooper PJ, Bradley M, Biswas G. The Global Programme to Eliminate Lymphatic Filariasis: health impact after 8 years. In: Institute of Medicine (US) Forum on Microbial Threats. The causes and impacts of neglected tropical and zoonotic diseases: opportunities for integrated intervention strategies. Washington, DC: National Academies Press, 2011.

[3] World Health Organization . Global Programme to Eliminate Lymphatic Filariasis: progress report 2022. Wkly Epidemiological Rec 2023; 41:489–502.

[4] King CL, Sumani J, Sanuku N, et al A trial of a triple-drug treatment for lymphatic filariasis. N Engl J Med 2018; 379:1801–10. 30403937 10.1056/NEJMoa1706854 PMC6194477

[5] Jambulingam P, Kuttiatt VS, Krishnamoorthy K, et al An open label, block randomized, community study of the safety and efficacy of co-administered ivermectin, diethylcarbamazine plus albendazole vs. diethylcarbamazine plus albendazole for lymphatic filariasis in India. PLoS Negl Trop Dis 2021; 15:e0009069. 33591979 10.1371/journal.pntd.0009069 PMC7909694

[6] Supali T, Djuardi Y, Christian M, et al An open label, randomized clinical trial to compare the tolerability and efficacy of ivermectin plus diethylcarbamazine and albendazole vs. diethylcarbamazine plus albendazole for treatment of brugian filariasis in Indonesia. PLoS Negl Trop Dis 2021; 15:e0009294. 33780481 10.1371/journal.pntd.0009294 PMC8031952

[7] Tavul L, Laman M, Howard C, et al Safety and efficacy of mass drug administration with a single-dose triple-drug regimen of albendazole + diethylcarbamazine + ivermectin for lymphatic filariasis in Papua New Guinea: an open-label, cluster-randomised trial. PLoS Negl Trop Dis 2022; 16:e0010096. 35139070 10.1371/journal.pntd.0010096 PMC8863226

[8] World Health Organization . Guideline: Alternative mass drug administration regimens to eliminate lymphatic filariasis. Geneva. 2017.

[9] Tripathi B, Roy N, Dhingra N. Introduction of triple-drug therapy for accelerating lymphatic filariasis elimination in India: lessons learned. Am J Trop Med Hyg 2022; 106:29–38. 35292580 10.4269/ajtmh.21-0964 PMC9154644

[10] World Health Organization . Global Programme to Eliminate Lymphatic Filariasis: Training in monitoring and epidemiological assessment of mass drug administration for eliminating lymphatic filariasis: Learners’ guide. WHO/HTM/NTD/PCT/2013.9. 2013.

[11] King CL, Weil GJ, Kazura JW. Single-dose triple-drug therapy for *Wuchereria bancrofti*—5-year follow-up. N Engl J Med 2020; 382:1956–7. 10.1056/NEJMc1914262 PMC7175637 32402169

[12] World Health Organization . Diagnostic test for surveillance of lymphatic filariasis to support decisions for stopping triple-therapy mass drug administration: target product profile. Geneva, Switzerland: World Health Organization, 2021.

[13] Won KY, Gass K, Biamonte M, et al Diagnostics to support elimination of lymphatic filariasis—development of 2 target product profiles. PLoS Negl Trop Dis 2021; 15:e0009968. 34780503 10.1371/journal.pntd.0009968 PMC8629375

[14] Sheel M, Sheridan S, Gass K, et al Identifying residual transmission of lymphatic filariasis after mass drug administration: comparing school-based versus community-based surveillance—American Samoa, 2016. PLoS Negl Trop Dis 2018; 12:e0006583. 30011276 10.1371/journal.pntd.0006583 PMC6062125

[15] Coffeng LE, Stolk WA, Golden A, de Los Santos T, Domingo GJ, de Vlas SJ. Predictive value of Ov16 antibody prevalence in different subpopulations for elimination of African onchocerciasis. Am J Epidemiol 2019; 188:1723–32. 31062838 10.1093/aje/kwz109 PMC6735885

[16] Stolk WA, Coffeng LE, Bolay FK, et al Comparing antigenaemia and microfilaraemia as criteria for stopping decisions in lymphatic filariasis elimination programmes in Africa. PLoS Negl Trop Dis 2022; 16:e0010953. 36508458 10.1371/journal.pntd.0010953 PMC9779720

[17] Irvine MA, Stolk WA, Smith ME, et al Effectiveness of a triple-drug regimen for global elimination of lymphatic filariasis: a modelling study. Lancet Infect Dis 2017; 17:451–8. 28012943 10.1016/S1473-3099(16)30467-4

[18] Subramanian S, Stolk W, Ramaiah K, et al The dynamics of *Wuchereria bancrofti* infection: a model-based analysis of longitudinal data from Pondicherry, India. Parasitology 2004; 128:467–82. 15180315 10.1017/s0031182004004822

[19] Jambulingam P, Subramanian S, de Vlas SJ, et al Mathematical modelling of lymphatic filariasis elimination programmes in India: required duration of mass drug administration and post-treatment level of infection indicators. Parasites Vectors 2016; 9:501. 27624157 10.1186/s13071-016-1768-y PMC5022201

[20] Behrend MR, Basáñez MG, Hamley JID , et al Modelling for policy: the five principles of the neglected tropical diseases modelling consortium. PLoS Negl Trop Dis 2020; 14:e0008033. 32271755 10.1371/journal.pntd.0008033 PMC7144973

[21] Michael E . The epidemiology of filariasis control. In: The Filaria. World Class Parasites. Vol 5. Boston, MA: Springer, 2002:59–74.

[22] Stolk WA, Subramanian S, van Oortmarssen GJ, Das PK, Habbema DJK. Prospects for elimination of bancroftian filariasis by mass drug treatment in Pondicherry, India: a simulation study. J Infect Dis 2003; 188:1371–81. 14593597 10.1086/378354

[23] Anderson R, May R. Population dynamics of human helminth infections: control by chemotherapy. Nature 1982; 297:557–63. 7088139 10.1038/297557a0

[24] Eneanya OA, Gankpala L, Goss CW, Bolay FK, Weil GJ, Fischer PU. Impact of annual versus semiannual mass drug administration with ivermectin and albendazole on helminth infections in southeastern Liberia. Am J Trop Med Hyg 2021; 106:700–9. 34814104 10.4269/ajtmh.21-0768 PMC8832944

[25] Prada JM, Davis EL, Touloupou P, et al Elimination or resurgence: modelling lymphatic filariasis after reaching the 1% microfilaremia prevalence threshold. J Infect Dis 2020; 221:S503–9. 31853554 10.1093/infdis/jiz647 PMC7289550

[26] World Health Organization . Guidelines for stopping mass drug administration and verifying elimination of human onchocerciasis: criteria and procedures. Geneva, Switzerland: Printed by the WHO Document Production Services, 2016. 26913317

[27] Subramanian S, Jambulingam P, Chu BK, et al Application of a household-based molecular xenomonitoring strategy to evaluate the lymphatic filariasis elimination program in Tamil Nadu, India. PLoS Negl Trop Dis 2017; 11:e0005519. 28406927 10.1371/journal.pntd.0005519 PMC5404881

[28] National Deworming Day Operational Guidelines . Child Health Division, Ministry of Health and Family Welfare, Government of India; 2015. Available at: https://nhm.gov.in/images/pdf/NDD/Guidelines/NDD_Operational_Guidelines.pdf . Accessed 4 January 2024.

[29] Pion SDS, Chesnais CB, Weil GJ, Louya F, Boussinesq M, Missamou F. Impact of semi-annual albendazole on lymphatic filariasis and soil-transmitted helminth infection: parasitological assessment after 14 rounds of community treatment. Am J Trop Med Hyg 2021; 106:729–31. 34929673 10.4269/ajtmh.21-0731 PMC8832930

[30] Pion SDS, Chesnais CB, Weil GJ, Fischer PU, Missamou F, Boussinesq M. Effect of 3 years of biannual mass drug administration with albendazole on lymphatic filariasis and soil-transmitted helminth infections: a community-based study in Republic of the Congo. Lancet Infect Dis 2017; 17:763–9. 28372977 10.1016/S1473-3099(17)30175-5

