# Supplementary material for: Predictive Value of Microfilariae-Based Stop-MDA Thresholds After Triple Drug Therapy With IDA Against Lymphatic Filariasis in Treatment-Naive Indian Settings
Source: Clin Infect Dis. 2024 Apr 25;78(Suppl 2):S131–7. doi: 10.1093/cid/ciae019 (PMC11045019; doi:10.1093/cid/ciae019)
Supplement: ciae019_Supplementary_Data [file ciae019_supplementary_data.pdf]

Supplement to:

The predictive value of microfilariae-based stop-MDA thresholds after triple drug therapy with IDA in treatment-naïve Indian settings

Ananthu James[1], Luc E. Coffeng[1], David J. Blok[1], Jonathan D. King[2], Sake J. de Vlas[1], Wilma A. Stolk[1]

[1] Department of Public Health, Erasmus MC, University Medical Center Rotterdam, Rotterdam, The Netherlands

[2] Department of Control of Neglected Tropical Diseases, World Health Organization, Geneva, Switzerland

Correspondence to: a.james@erasmusmc.nl

Figures

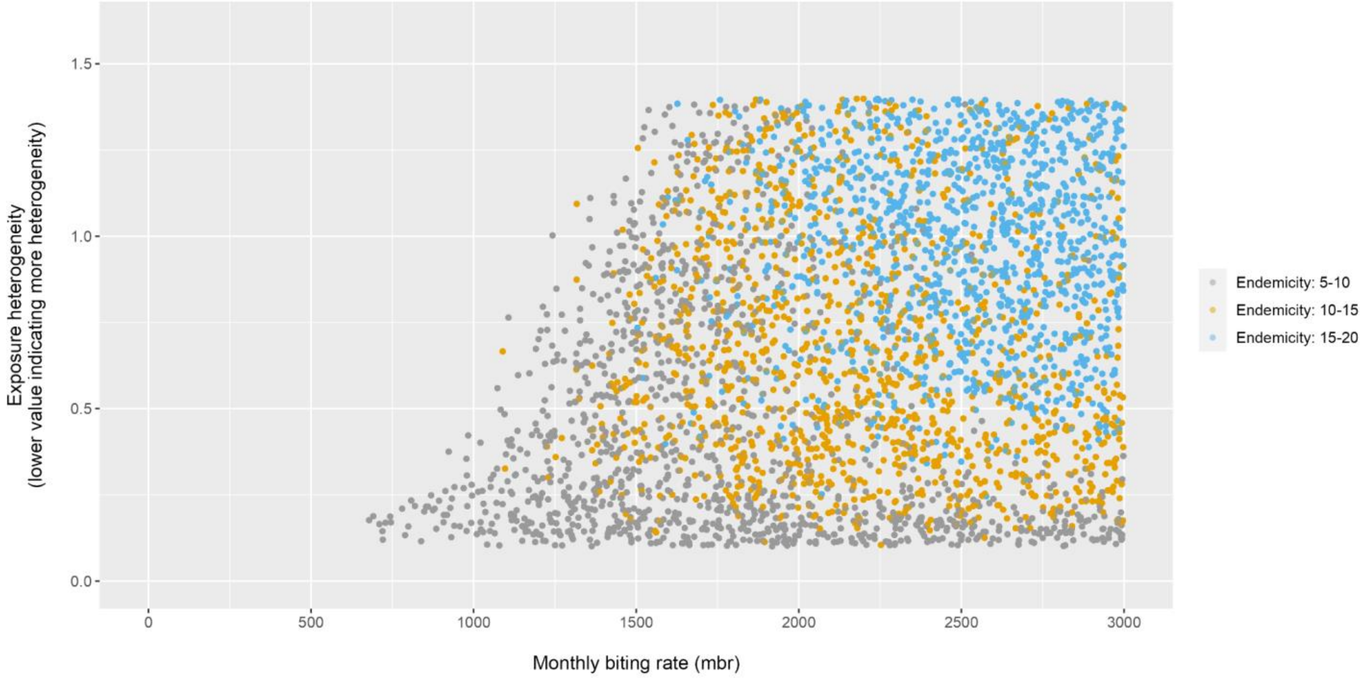

Figure S1. Parameter combinations used for simulations, coloured by baseline endemicity class, with endemicity defined in terms of mf prevalence. Note that these endemicity classes do not appear in the results, since all the simulations in this article are performed after lumping endemicity classes together.

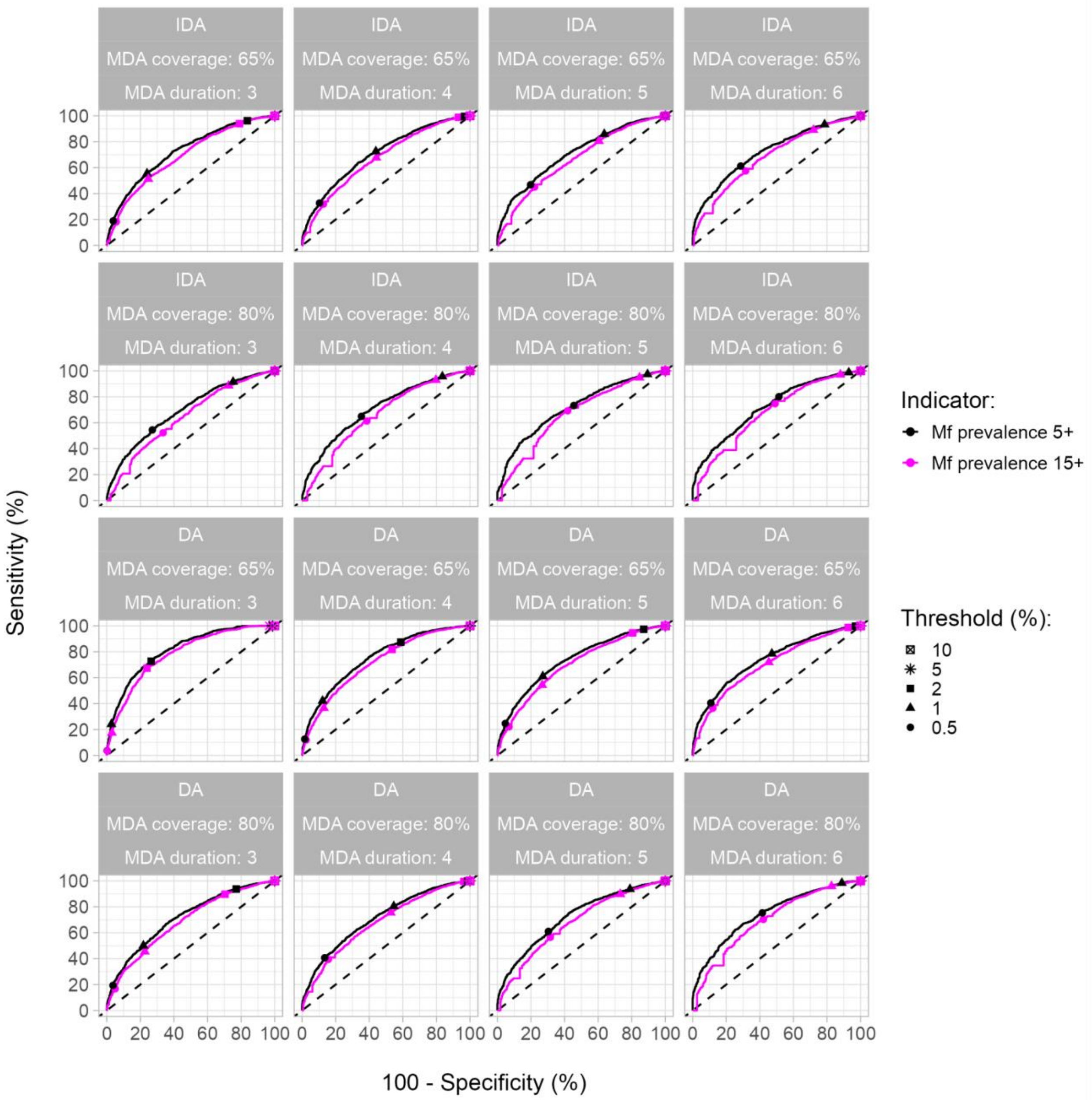

Figure S2. Receiver-operator characteristic (ROC) curves for two different age groups 1 year post-MDA (TAS-1), when elimination was defined within 20 years post-MDA. The panels in the top two rows represent MDA with IDA and the bottom ones MDA with DA.

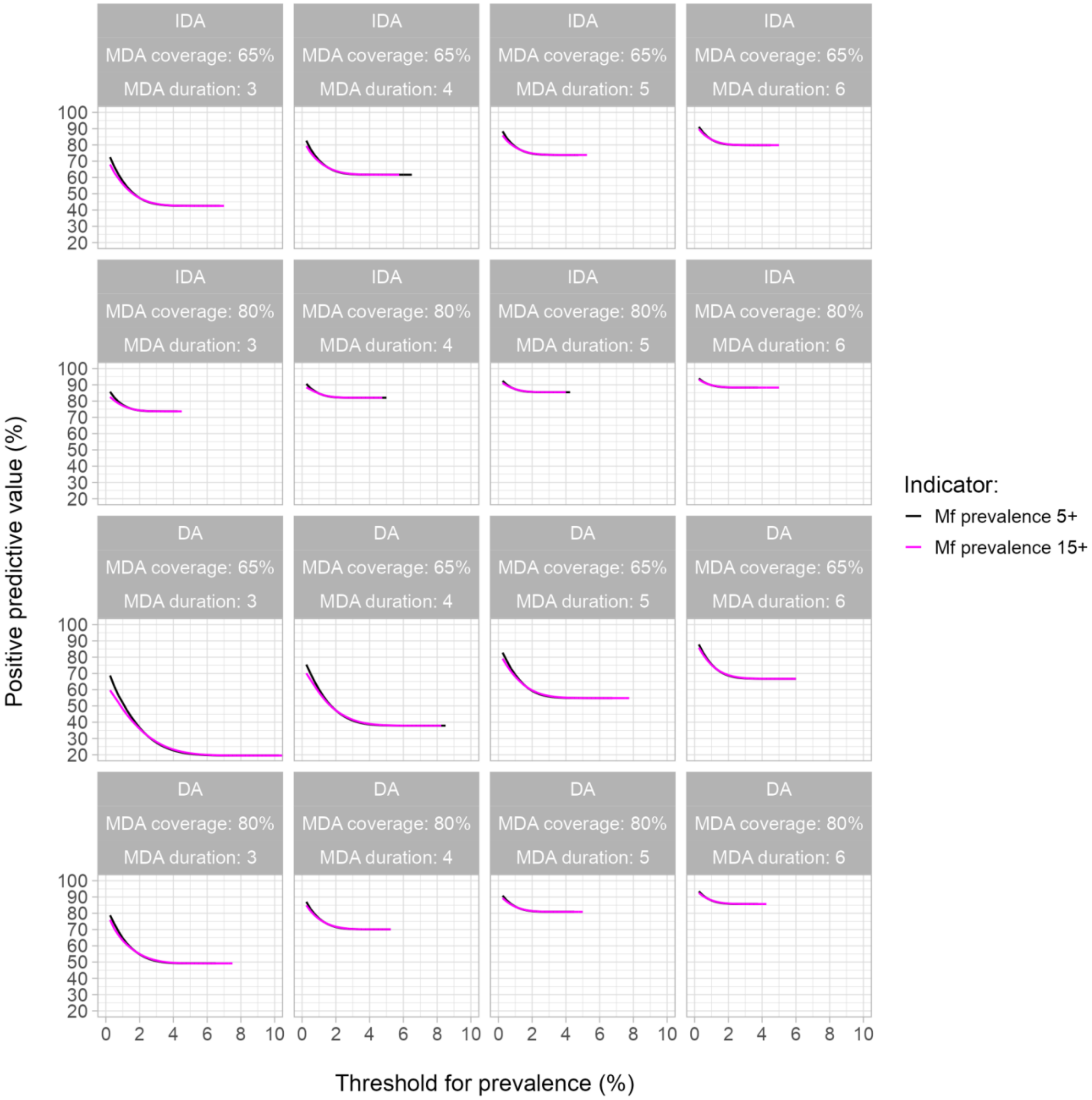

Figure S3. Positive predictive value (PPV) of TAS-1 for elimination of LF for different values of MDA duration and coverage. Elimination was defined as zero mf prevalence 20 years after the last MDA round. PPVs were calculated as a function of the stop-MDA threshold for prevalence of infection (horizontal axis) in the age groups 5+ (black curve) and 15+ (magenta curve). The panels in the top two rows represent MDA with IDA and the bottom two rows represent MDA with DA. We assumed a sample size of 400.

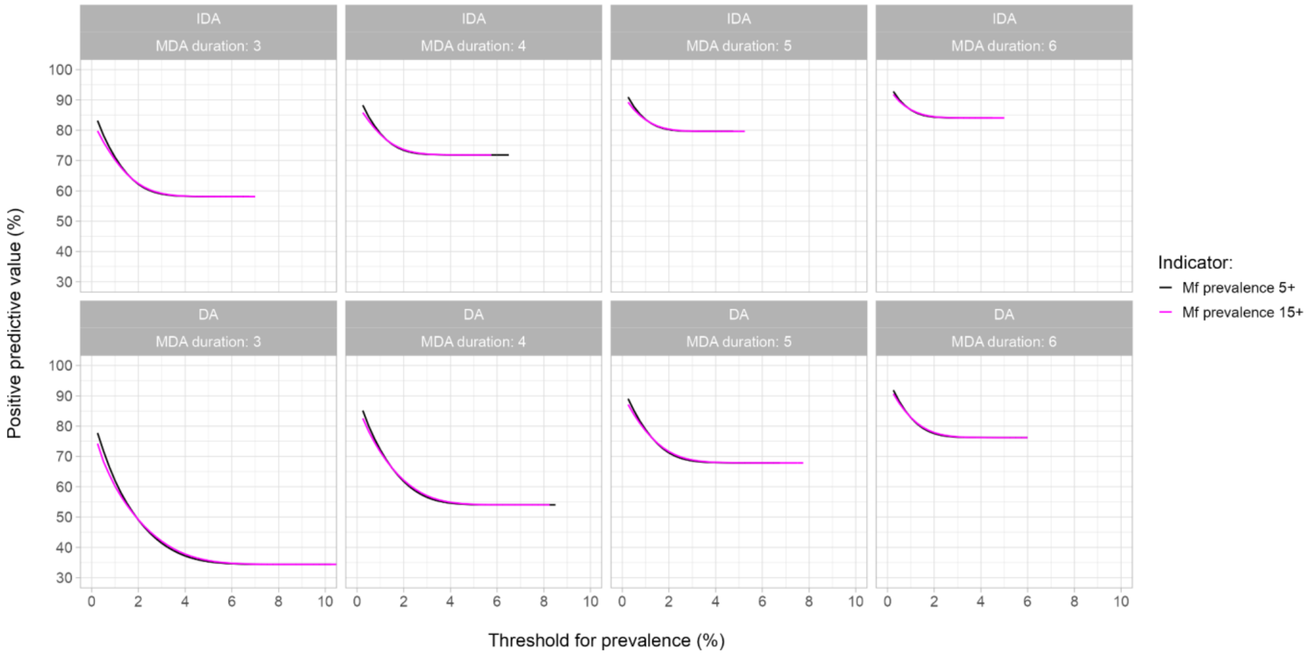

Figure S4. Positive predictive value (PPV) of TAS-1 for elimination of LF for different values of MDA duration when coverages were lumped together for different age groups. Elimination was defined within 20 years post-MDA. PPVs were calculated as a function of the stop-MDA threshold for prevalence of infection (horizontal axis) in the age groups 5+ (black curve) and 15+ (magenta curve). The top panels represent MDA with IDA and the bottom panels represent DA. The sample size was 400.

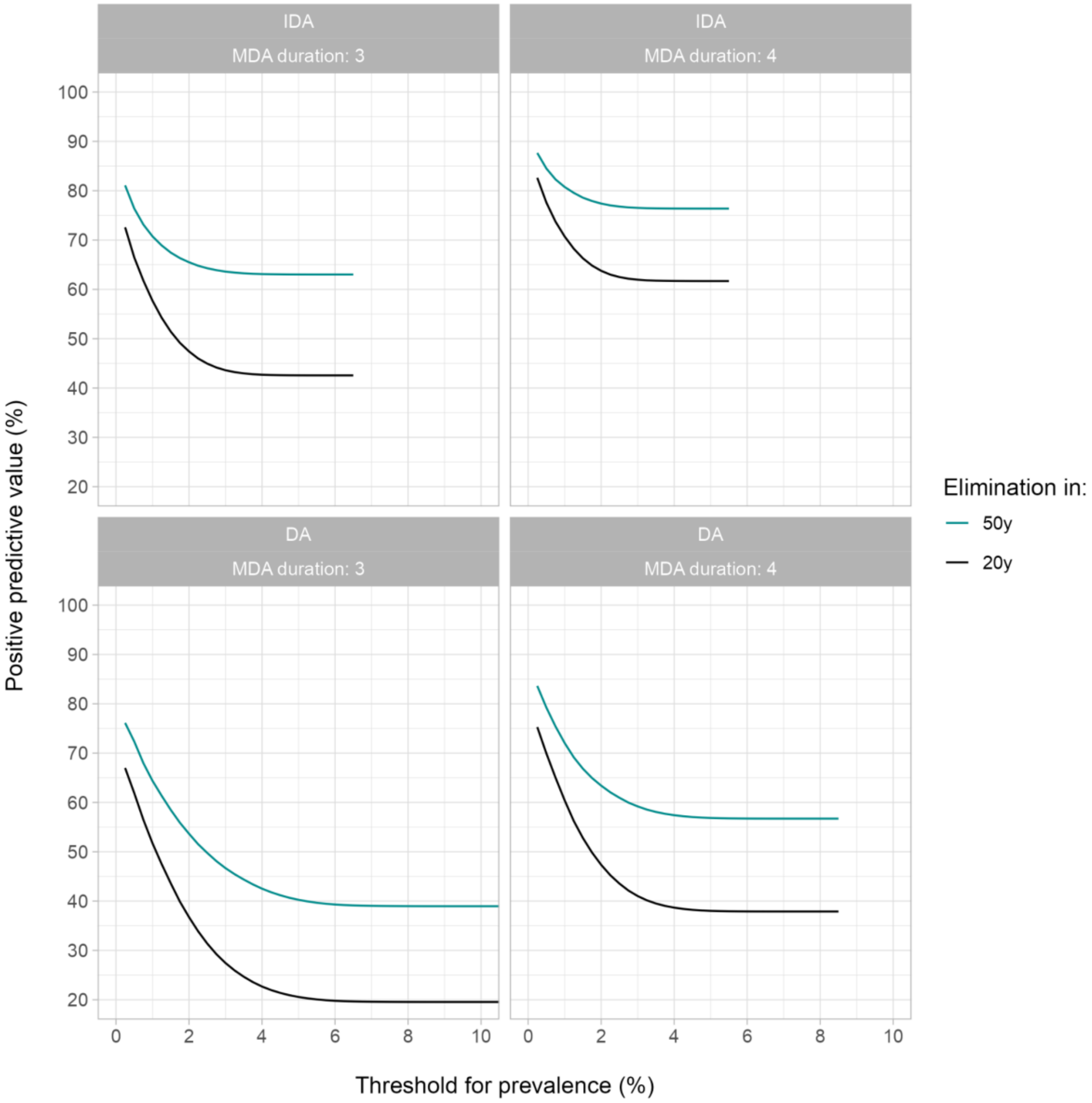

Figure S5. Positive predictive value (PPV) of TAS-1, when elimination was defined within 20 and 50 years post-MDA, for 65% MDA coverage. The different panels show two different drug regimens and MDA durations. PPVs were calculated as a function of the stop-MDA threshold for prevalence of infection (horizontal axis) in the age groups 5+. The top panels represent MDA with IDA and the bottom ones represent DA. The sample size was 400.

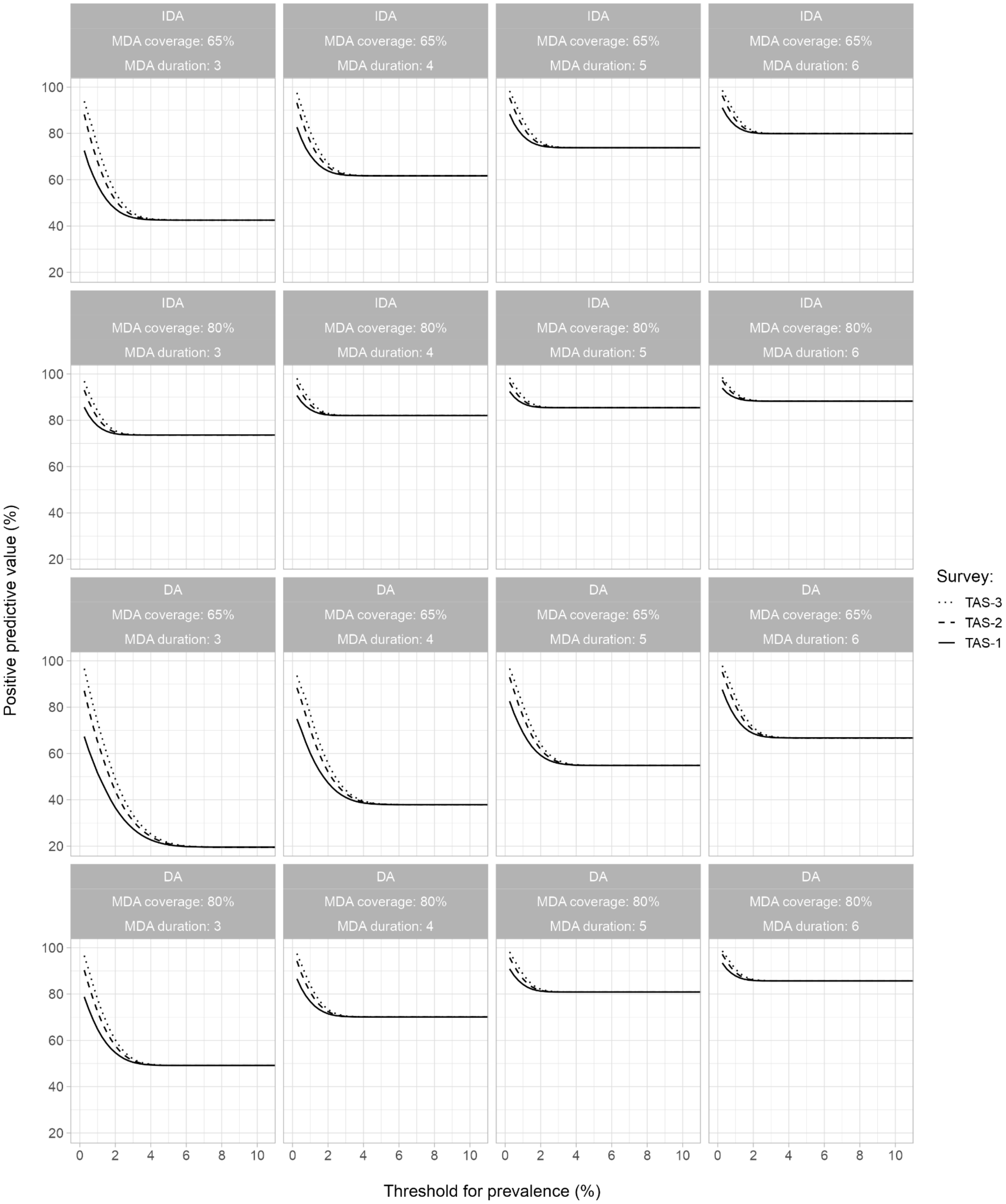

Figure S6. Positive predictive value (PPV) of TAS-1, TAS-2, and TAS-3 for elimination of LF for different values of MDA duration and coverage. Elimination was defined as zero mf prevalence 20 years after the last MDA round. PPVs were calculated as a function of the stop-MDA threshold for prevalence of infection in the age group 5+ (horizontal axis). For TAS-2 and TAS-3, PPVs are conditional on all previous TAS-es being passed with the same prevalence threshold. TAS-1, 2, and 3 were scheduled 1, 3, and 5 years post-MDA, so that the gap between consecutive TAS-es was 2 years. The panels in the top two rows represent MDA with IDA and the bottom two represent DA. For each TAS, we assumed a sample size of 400.

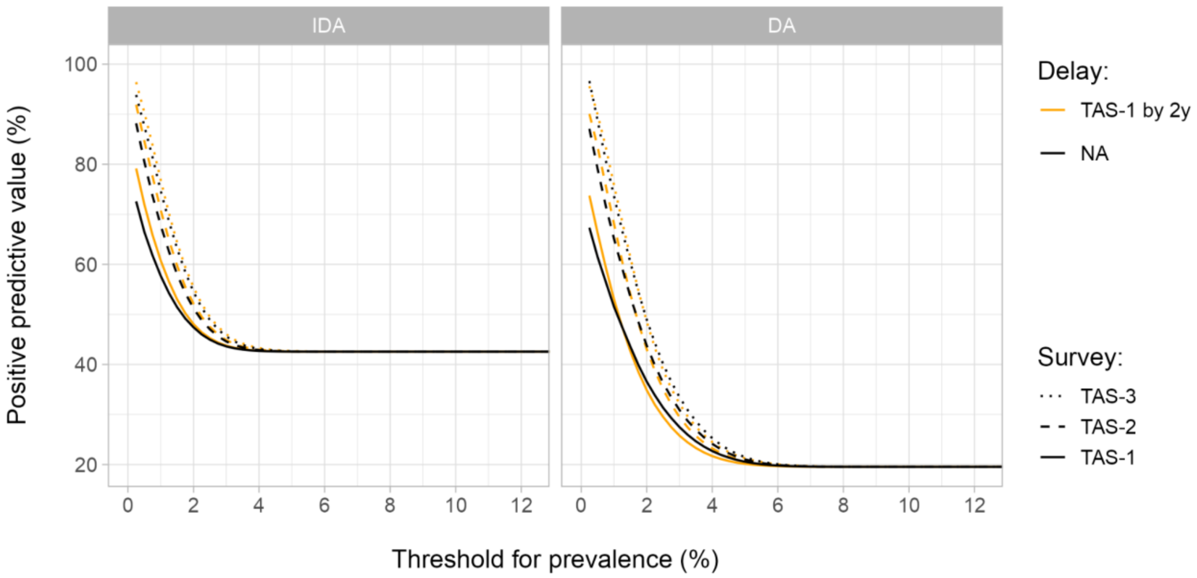

Figure S7. Positive predictive value (PPV) of TAS-1, TAS-2, and TAS-3 for elimination of LF following MDA for 3 years at 65% coverage. Elimination was defined as zero mf prevalence 20 years after the last MDA round. PPVs were calculated as a function of the stop-MDA threshold for prevalence of infection in the age group 5+ (horizontal axis). For TAS-2 and TAS-3, PPVs are conditional on all previous TAS-es being passed with the same prevalence threshold. We considered two situations for the surveys: (i) TAS-1, 2, and 3 being scheduled 1, 3, and 5 years post-MDA (black curves) and (ii) TAS-1, 2, and 3 being scheduled 3, 5, and 7 years post-MDA (dark yellow curves). The left panel represents MDA with IDA and the right one represents DA. For each TAS, we assumed a sample size of 400.

Table S1. PRIME-NTD table: Policy-Relevant Items for Reporting Models in Epidemiology of Neglected Tropical Diseases

Principle	What has been done to satisfy the principle?	Where in the manuscript is this described?
1. Stakeholder engagement	The work was initiated following a request from Jonathan D. King at the World Health Organization (WHO), Geneva. He was constantly updated at various stages of this work, and he is also a co-author of this paper. Further, this work has been presented at the Annual Meeting of NTD Modelling Consortium (NTDMC) held at Oxford in April 2023, where stakeholders from WHO as well as the Bill and Melinda Gates Foundation (BMGF) were present.	Author list
2. Complete model documentation	We used a previously published model, the details of which are available elsewhere (relevant sources cited). Further, we have provided the full description of any additional aspect, including parameter quantifications such as baseline mf prevalence, drug efficacy, and MDA duration, coverage, and frequency, considered in our model.	Methods section and Fig. S1 (in the Supplement)
3. Complete description of data used	The data relevant for this work were quantified and described in detail in previous publications. These articles were mentioned in the Methods.	Methods section
4. Communicating uncertainty	We have considered parameter variation across MDA duration and coverage, in different age groups (5+ and 15+). We have also used two different sample sizes (200 and 400) for the TAS-es as well as two different time periods (20y and 50y) to reach elimination to assess the sensitivity.	Results and Figs. S2-S7.
5. Testable model outcomes	All the model predictions are testable, provided the relevant data are collected. With the shorter 20y period for elimination considered here, with regard to the previous articles that used a 50y period, it is more feasible that the PPVs here can be tested against results from actual settings.	Fig. S5 and Discussion
